# Evolution in endoscopic endonasal approach for the management of hypothalamic–pituitary region metastasis: A single-institution experience

**DOI:** 10.3389/fonc.2022.975738

**Published:** 2022-07-28

**Authors:** Cinzia Baiano, Teresa Somma, Raduan Ahmed Franca, Marianna Di Costanzo, Maria Rosaria Scala, Pasquale Cretella, Felice Esposito, Luigi Maria Cavallo, Paolo Cappabianca, Domenico Solari

**Affiliations:** ^1^ Division of Neurosurgery, Department of Neurosciences, Reproductive and Odontostomatological Sciences, Università degli Studi di Napoli “Federico II”, Naples, Italy; ^2^ Pathology Section, Department of Advanced Biomedical Sciences, Università degli Studi di Napoli “Federico II”, Naples, Italy

**Keywords:** hypothalamic–pituitary pathology, endoscopic endonasal surgery, brain metastasis, neuro-oncology-surgical, surgical procedures

## Abstract

**Introduction:**

Endonasal endoscopic surgery has changed the treatment perspectives for different lesions of the hypothalamic–pituitary region. The metastases of the hypothalamic–pituitary region represent 0.4% of all intracranial metastatic tumors and account for only 1.8% of surgically managed pituitary lesions. The aim of tshis study is to describe a single-center institutional experience with 13 cases of hypothalamic–pituitary metastasis focused on presurgical workup, the evolution of the surgical technique, and postsurgical management according to our protocols, showing effects on progression-free and overall survival rates for this relatively uncommon location.

**Material and Methods:**

We retrospectively reviewed the whole series of patients that received the endoscopic endonasal approach at the Division of Neurosurgery at the University of Naples “Federico II” undergoing surgery from January 1997 to December 2021. We identified 13 cases whose pathology reports revealed a metastatic lesion. Statistical analysis was performed to determine the Kaplan–Meier survival function and assess for log-rank differences in survival based on gender, surgical treatment, and postoperative therapy (*p*-value < 0.02*).

**Results:**

The pathology report disclosed lung adenocarcinoma (six cases, 46%), breast adenocarcinoma (two cases, 15.4%), clear cell renal carcinoma (one case, 7%), melanoma (one case, 7%), colorectal adenocarcinoma (one case, 7%), uterine cervix carcinoma (one case, 7%), and follicular thyroid carcinoma (one case, 7%). A standard endoscopic endonasal approach was performed in 10 patients (76.9%), while an extended endonasal procedure was performed in only three cases (23%). Biopsy was the surgical choice in five patients with infiltrative and invasive lesions and a poor performance status (38%), while in the cases where neurovascular decompression was necessary, a subtotal resection was achieved in five patients (38%) and partial resection in three patients (23%). Recovery of visual field defect was observed in six of seven patients with visual loss (85.7%), improvement of oculomotor nerve palsy occurred in four of seven patients with this defect (57.1%), while the impairment of oculomotor palsy was observed in three patients (42.9%). Visual function was stable in the other patients. The median progression-free survival and overall survival were 14 and 18 months, respectively. There were statistically significant differences in PFS and OS in patients who underwent adjuvant radiotherapy (p=0.019 is referred to OS and p=0.017 to PFS, respectively; *p*-value = 0.02).

**Conclusions:**

The endoscopic endonasal approach is a viable approach for the management of hypothalamic–pituitary metastases as this surgery provides an adequate opportunity to obtain tissue sample and neurovascular decompression, both being crucial for continuing the integrated adjuvant therapy protocols.

## Introduction

The inherent characteristics of endonasal endoscopic surgery have changed the treatment perspectives and operative nuances for different lesions of the hypothalamic–pituitary region (1).

This surgical route, in the standard version, has provided a more accurate distinction between healthy and pathological tissues in pituitary micro- and macroadenoma removal, thus ensuring pituitary gland preservation, an increase in the extent of resection, and an accurate histopathological characterization. Then, the extended approach, through access to the suprasellar, parasellar, retrosellar, clival, and retroclival spaces by shorter surgical corridors than the transcranial route, has revolutionized the management of complex and non-adenomatous midline lesions such as craniopharyngiomas, meningiomas, and clival chordomas (2–5). For the less common and more infiltrating lesions such as sarcomas, gliomas, metastases, and granulomatosis (sarcoidosis), it has enabled a minimally invasive biopsy and/or the identification of surgical removal limits, taking into consideration the principles of maximal safe resection (6).

The metastases of the hypothalamic–pituitary region represent 0.4% of all intracranial metastatic tumors and account for only 1.8% of surgically managed pituitary lesions (7). Metastatic tumor cells may involve the pituitary gland *via* different patterns of spread, including direct hematogenous, from the hypothalamus or stalk through the portal hypophyseal vessels or from the juxtasellar or skull base metastasis through the arachnoid of the suprasellar cistern (8, 9).

At our institution, in a dedicated tertiary center for hypothalamic–pituitary disorders, the possibility of observing more than one hundred endoscopic endonasal procedures per year has granted the wide series and also the variety with the inclusion of metastatic pathology of the aforementioned region (10–12).

In the circular process of update application in clinical complementary fields adjacent to the midline skull base surgery (endocrinology, neuroradiology, pathology, radiotherapy, and oncology), the new strategies of approach to hypothalamic–pituitary lesions are dependent on a multidisciplinary approach (13–18). Furthermore, advancements in target systemic radiosurgery and whole-brain radiotherapy and therapy for brain metastasis management have changed prognostic models (19–22). In this setting, the endoscopic endonasal approach is proposed as a valid tool for obtaining tissue for histological examination and determining consequently therapeutical steps in the treatment of metastatic patients (23–25).

The aim of this study is to describe a single-center institutional experience with 13 cases of sellar metastasis focused on presurgical workup, evolution of surgical technique, and postsurgical management according to our protocols, showing effects on progression-free and overall survival rates for this relatively uncommon location.

## Methods

This study was approved by the Institutional Review Board of the School of Medicine of the University of Naples “Federico II,” which waived the need for informed consent due to the retrospective nature of the study. Written informed consent was obtained from the patients prior to any invasive clinicodiagnostic and surgical procedure; indeed, it was obtained for the eventual publication—for scientific purposes—of any patient records/information anonymously.

We retrospectively reviewed the whole series of patients that received the endoscopic endonasal approach at the Division of Neurosurgery at the University of Naples “Federico II” undergoing surgery from January 1997 to December 2021. We identified 13 cases whose pathology report revealed a metastatic lesion.

Case history, histological diagnosis, endocrinological assessment, preoperative and postoperative radiological records, preoperative treatment, intraoperative surgical videos, and instrumental eye examinations were revised. All patients underwent a pituitary pre- and postsurgical function assessment, pre- and postoperative post-gadolinium magnetic resonance (MRI), and complete visual assessment (computerized visual field, Lancaster red-green test, visual acuity).

**Figure 1:**
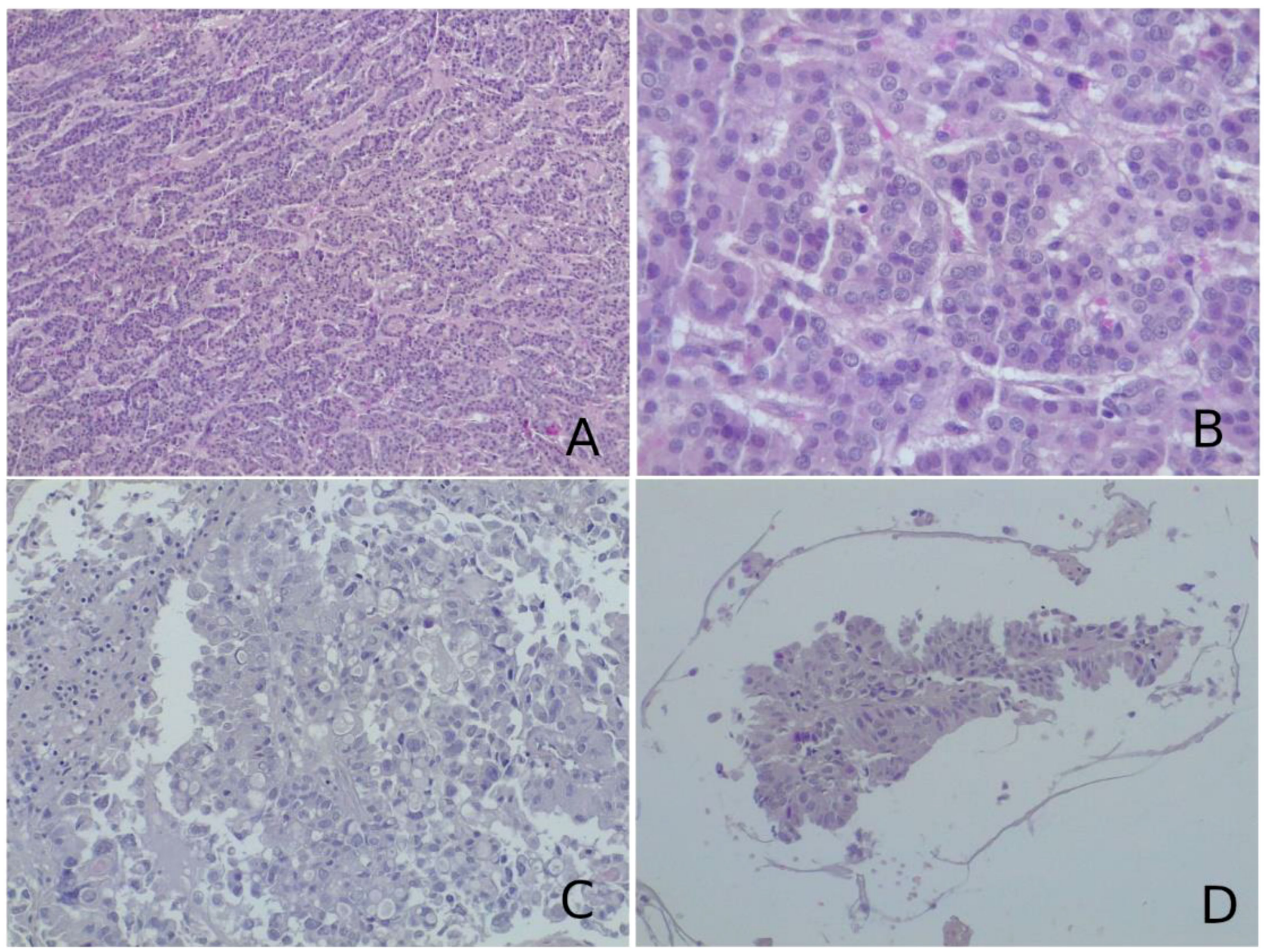
**(A, B)** Histological examination of the lesion biopsied revealed a neoplasm composed of cells arranged in follicular, cord-like, and nodular structures. On immunohistochemistry, neoplastic cells were positive for thyroglobulin, PAX8, TTF1, and cytokeratin 19 and negative for CD56 and CDX2. This morphological picture was suggestive of metastatic follicular thyroid carcinoma. **(C, D)** On histological slides, a glandular neoplasm, composed of pleomorphic, vacuolated cells with high-grade characteristics and papillary arrangement [most clear in **(D)**], was seen. Neoplastic cells were immunoreactive for cytokeratin 7 and TTF1 and negative for cytokeratin 20. These characteristics were more suggestive of adenocarcinoma metastasis from a lung primary tumor. **(A–D)** Hematoxylin–eosin, original magnification ×40.

Statistical analysis was performed to determine the Kaplan–Meier survival function and assess for log-rank differences in survival based on gender, surgical treatment, and postoperative therapy (*p*-value < 0.02). All analyses were performed using the R environment software for statistical computing (**Figure 1**) (R Development Core Team, Vienna, Austria, 2013).

### Surgical Technique

All the patients underwent an endoscopic endonasal approach. For intrasellar lesion, a standard operative nuance was performed, while in the case of supradiaphragmatic lesion, the most suitable extended approach was chosen according to the techniques already described (2). In all cases, extemporaneous histological examination was decisive for subsequential surgical steps.

### Pathology

Unusual morphological patterns can cause diagnostic concern for other lesions (sinusoidal pattern and macronodular or festoon-like features, as well as lesions with diffuse epithelioid features). In these cases, a routine immunohistochemical panel was performed (ACTH, PRL, GH, TSH, FSH, LH, GH, Ki67), including reticulin staining, neuroendocrine differentiation using immunohistochemical markers (chromogranin, synaptophysin), and immunoreactivity for transcription factors (T-Pit, Pit1, SF1), which were directed toward a correct diagnosis.

The differential diagnosis between a pituitary adenoma and metastatic cancer is rarely a problem: mitotic activity and cellular pleomorphism are nearly always the hallmarks of a metastatic neoplasm, while these are rare in pituitary adenomas.

When a lesion is thought to be a metastasis with no primary tumor clearly diagnosed, additional markers should be performed to define the tumor lineage: LCA (CD45) positivity is seen in lymphoproliferative lesions; carcinomas are nearly always pan-cytokeratin-positive; TTF1 positivity suggests a pulmonary or thyroid origin (the latter being positive also for thyroglobulin and PAX8); CDX2 positivity suggests cancer originating in the gastroenteric tract; HMB45 along with MART1, SOX10, and S100 immunoreactivity is a feature of melanoma; GCDFP-15 and mammaglobin are markers of breast cancer; PSA positivity suggests a prostate primitivity; and PAX8 immunoreactivity supports a diagnosis of metastatic renal cell carcinoma.

**Figure 2:**
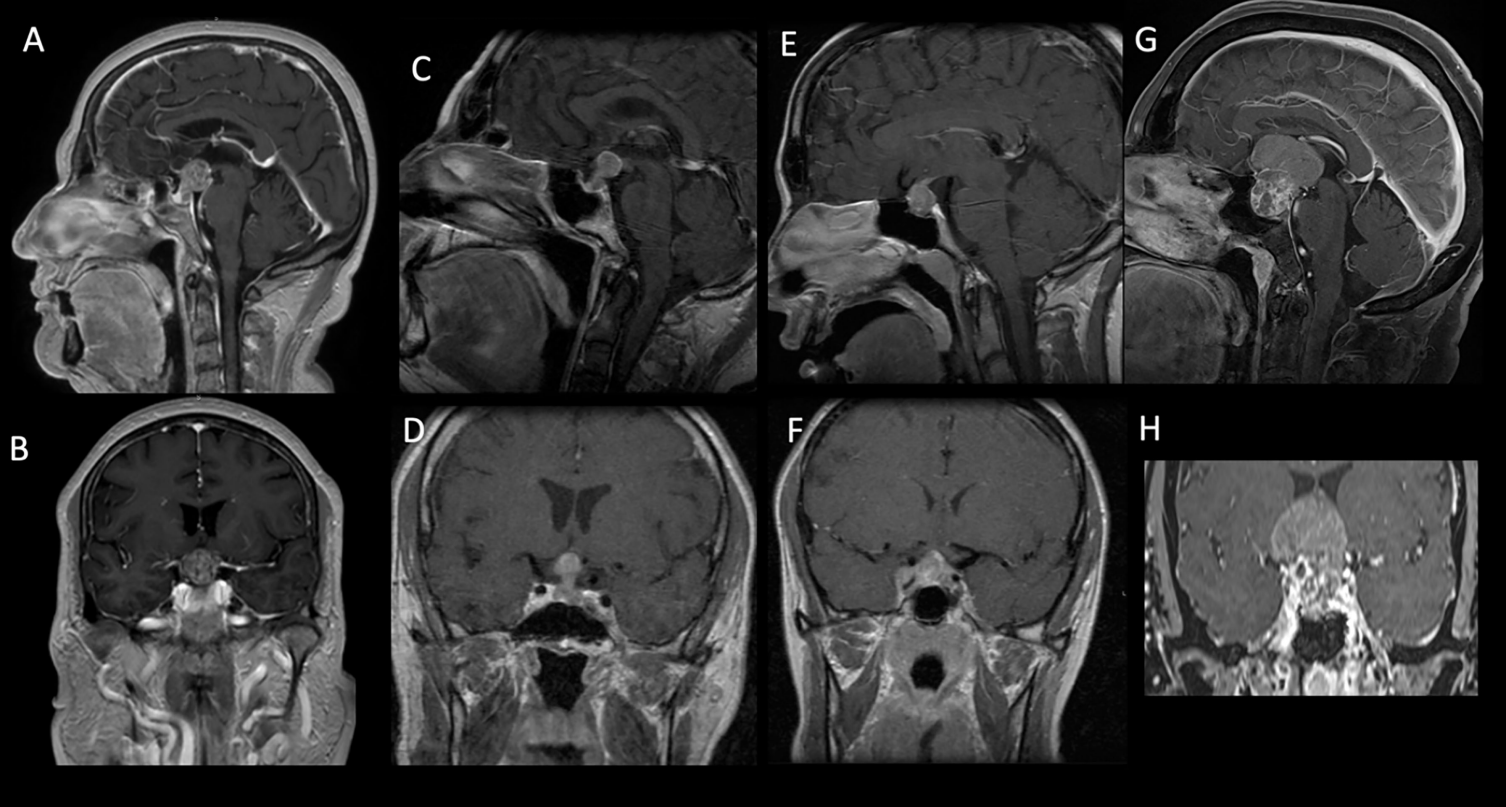
Sagittal and coronal preoperative MRI T1-weighted contrast-enhanced images of patients from our cohort showing intra- and suprasellar lesions with inhomogeneous enhancement. The pathology report disclosed breast adenocarcinoma **(A**, **B)**, lung adenocarcinoma **(C**, **D)**, clear cell renal carcinoma **(E**, **F)**, and uterine cervix carcinoma (**G, H**).

A challenging differential diagnosis concerns distinguishing null cell adenoma from metastatic neuroendocrine carcinoma: indeed, both are tumors immunonegative for all the hypophysial markers (hormones and transcription factors) but positive for neuroendocrine markers. Morphology, mitotic activity, and lineage differentiation markers (TTF1, CDX2, CK, calcitonin, etc.) are useful to make a correct diagnosis. However, it must be kept in mind the possibility of the debated entity of primary intracranial neuroendocrine carcinoma arising in the sellar region (TTF1−) and small cell carcinoma of unknown primary (SCUP) (TTF1+) (**Figure 2**).

## Results

Between around 1997 and December 2021, 2,303 patients underwent endonasal endoscopic surgery for the removal of different skull base lesions—mostly pituitary adenomas—at the Division of Neurosurgery at the University of Naples “Federico II.” Of these patients, 13 (0.6%) had a metastatic lesion (**Table 1**). Nine patients were women and four were men; the mean age was 58 years. Three patients presented a pure infradiaphragmatic intrasellar lesion (23%); four patients presented intra-, supra-, and parasellar lesions (30%); and six patients had intra-, supra-, and retrosellar lesions (46%). The most common presentation was headache in 10 patients (76.9%), followed by visual loss in 7 patients (53.8%), adenohypophysis dysfunction in 6 patients (46%), diabetes insipidus in 6 patients (46%), visual field defect in 3 patients (23%), and oculomotor nerve palsy in 3 patients (23%) (**Table 2**). In the five cases of lung adenocarcinoma, pituitary metastasis was the first presentation of neoplastic disease.

**Table 1:** Patient demographics and primary neoplasm.

Patient	Age (years)	Sex	Cancer type	Known metastatic disease
**1**	45	M	Lung adenocarcinoma	−^a^
**2**	48	F	Lung adenocarcinoma	−^a^
**3**	68	F	Melanoma	+
**4**	53	F	Breast carcinoma	+
**5**	59	F	Lung adenocarcinoma	−^a^
**6**	68	M	Squamous cell carcinoma (lung)	+
**7**	65	F	Lung adenocarcinoma	−^a^
**8**	50	F	Cervical carcinoma	+
**9**	62	F	Follicular thyroid carcinoma	+
**10**	67	F	Colorectal adenocarcinoma	+
**11**	80	M	Clear cell renal carcinoma	+
**12**	65	F	Breast carcinoma	+
**13**	51	M	Lung adenocarcinoma	−^a^

a Pituitary metastasis was the first presentation of neoplastic disease.

**Table 2:** Symptoms at presentation.

Patient	Cranial nerve palsy (3, 4, 6)	Visual field defect	Adenohypophyseal dysfunction	Diabetes insipidus	Headache
**1**	−	BT	Hypothyroidism, hypercortisolism	−	+
**2**	+ (3, 6, 4)	−	Hypocortisolemia, hypogonadism	+	−
**3**	+ (3)	−	−	−	−
**4**	−	BT	−	+	+
**5**	+ (3, 6)	−	−	−	+
**6**	−	−	−	−	+
**7**	−	−	Hypergonadism, hypocortisolism	+	−
**8**	−	+	Hypercortisolemia	+	+
**9**	+	+	Hypothyroidism		+
**10**	−	−	/	−	+
**11**	+	+	−	+	+
**12**	+	+	−	−	+
**13**	+ (3)	+	Hypocortisolemia, hypogonadism	+	+

(3), 3rd cranial (oculomotor) nerve; (4), 4th cranial (trochlear) nerve; (6), 6th cranial (abducens) nerve; BT, bitemporal hemifield defect.

The surgical strategy was tailored based on the lesion extension and the performance status of the patients: in three cases, an extended endonasal approach was required; in five cases, only a biopsy was performed. The endoscopic endonasal standard approach was used in 10 patients; for the other three patients, an extended trans-planum approach was performed. Osteodural defect reconstruction was necessary in two cases (**Table 3**).

The pathology report disclosed lung adenocarcinoma (six cases, 46%), breast adenocarcinoma (two cases, 15.4%), clear cell renal carcinoma (one case, 7%), melanoma (one case, 7%) colorectal adenocarcinoma (one case, 7%), uterine cervix carcinoma (one case, 7%), and follicular thyroid carcinoma (one case, 7%) (**Table 1** and **Figure 1**).

The standard endoscopic endonasal approach was performed in 10 patients (76.9%), while the extended endonasal procedure was used in only three cases (23%). Biopsy was the surgical choice in five patients with infiltrative and invasive lesions and a poor performance status (38%). On the other hand, in the cases where neurovascular decompression was necessary, a subtotal resection was achieved in five patients (38%) and a partial resection in three patients (23%).

Recovery of visual field defect was observed in six of seven patients with visual loss (85.7%), improvement of oculomotor nerve palsy occurred in four of seven patients with this defect (57.1%), while impairment of oculomotor palsy was observed in three patients (42.9%). Visual function was stable in the other patients.

Concerning complications, no infection and CSF leak were seen; we observed one patient developing transient diabetes insipidus.

Adjuvant therapy was used in all cases. Ten patients were treated with systemic chemotherapy (59%), two patients (20%) had stereotactic radiotherapy, and one patient with a clear cell renal carcinoma had a combination of radio- and chemotherapy. After adjuvant treatment, three patients (23%) developed pan-hypopituitarism.

**Figure 3:**
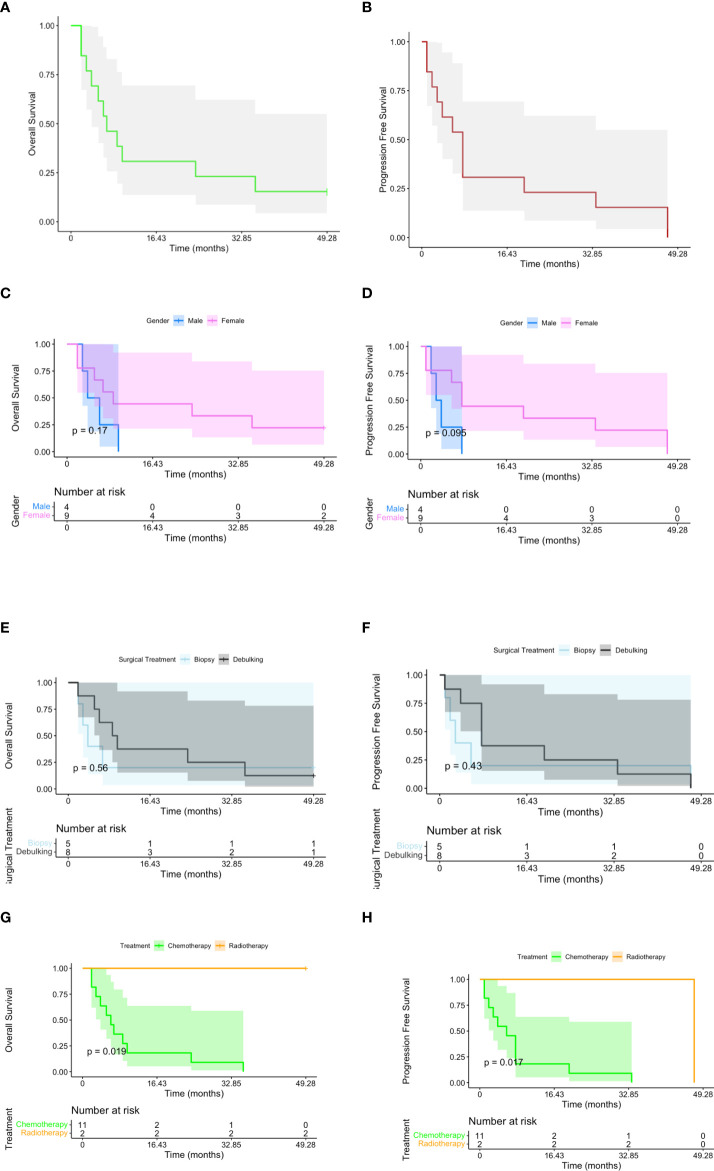
Kaplan–Meier plots reporting overall survival (OS) and progression-free survival (PFS) for all the subjects included in the study **(A**, **B)**. OS and PFS have been stratified, respectively, by gender **(C**, **D)**, surgical treatment **(E**, **F)**, and postoperative therapy [**G** (*p*-value < 0.02), **H** (*p*-value < 0.02 )].

The median progression-free survival and overall survival were 14 and 18 months, respectively (**Table 4**). There were statistically significant differences in PFS and OS in patients who underwent adjuvant radiotherapy (p=0.019 is referred to OS and p=0.017 to PFS, respectively; *p*-value = 0.02) (**Figure 3**).

**Table 4:** Surgical and adjuvant management.

Patient	Surgery (EEA)	Adjuvant therapy	Progression-free survival	Overall survival
**1**	Debulking	Chemotherapy	8 months	10 months
**2**	Biopsy	Chemotherapy	6 months	7 months
**3**	Debulking (partial)	Chemotherapy	8 months	9 months
**4**	Debulking	Chemotherapy	34 months	36 months
**5**	Debulking (partial)	Chemotherapy	1 month	2 months
**6**	Biopsy	Chemotherapy	3 months	4 months
**7**	Biopsy	Chemotherapy	1 month	2 months
**8**	Debulking (partial)	Radiotherapy	48 months	Alive
**9**	Biopsy	Radiotherapy	48 months	Alive
**10**	Debulking	Chemotherapy	8 months	NA
**11**	Biopsy	Radiotherapy + chemotherapy	2 months	3 months
**12**	Debulking	Chemotherapy	20 months	2 years
**13**	Debulking	Chemotherapy	4 months	6 months

EEA, endoscopic endonasal approach; NA, not available.

## Discussion

Hypothalamic–pituitary metastatic lesions represent a very challenging diagnosis: clinical signs are not different from the other lesions affecting this area, and there are no pathognomonic signs at MRI or CT scan (6, 26). Moreover, the lack of a certain presence of tumor history jeopardizes the likelihood of a lesion of the hypothalamic–pituitary region being a metastasis (9). In the present series, we found five cases of hypothalamic–pituitary metastasis figured out as the first lesion of neoplastic disease.

A positive oncological anamnesis for the most common primary tumors associated with hypothalamic–pituitary metastasis (breast cancer in women, 40% of the cases; lung cancer in men, 24% of the cases) could verify the suspicion. Other primary tumors that are less common include gastrointestinal tract (6.3%), prostate (5%), melanoma (2.4%), and thyroid (2.2%) malignancies (8, 23, 27). Primitive tumors observed in this series were lung adenocarcinoma (six cases, 46%), breast adenocarcinoma (two cases, 15.4%), clear cell renal carcinoma (one case, 7%), melanoma (one case, 7%), colorectal adenocarcinoma (one case, 7%), cervical carcinoma (one case, 7%), and follicular thyroid carcinoma (one case, 7%) (**Table 1**).

In agreement with the current literature (8, 27–30), the main clinical manifestation was headache (76.9%), followed by visual loss (53.8%), adenohypophysis dysfunction (46%), diabetes insipidus (46%), visual field defect (23%), and oculomotor nerves palsy (23%). In this scenario, we have to consider diabetes insipidus as a typical feature of infiltrative non-adenomatous sellar lesions (sarcoidosis, hypophysitis, histiocytosis, craniopharyngiomas, ATRT) and, above all, when it is associated with visual loss and/or nerve palsy (6, 31).

Microsurgical trans-sphenoidal, open transcranial, and trans-facial surgery were the last common surgical routes to be reported in the management of pituitary metastasis, and the mortality and morbidity related to these procedures were not insignificant (28, 32).

Initially, the endoscopic endonasal technique was considered a two-handed technique, and similar to the microsurgical technique, the basic principles used are not far from those when operating using a microscope. The evolution of the four-handed technique, in a setting of close and dynamic cooperation between two operators, has granted a useful amplification of the surgical corridor and the augmentation of the angle of exposure by a continuous change of framing, focus, and distance view. The increase in surgical agility, allowing improvement in the dissection method, has made the endoscopic endonasal technique a valid tool for managing the entire ventral skull base, according to the same surgical principles of the open approaches, offering a possibility of treating a wide variety of median and paramedian lesions with satisfactory outcomes (2, 5, 33, 34).

The possibility to access the supra-, para-, and retrosellar spaces by extended procedures has changed the way of exploring and removing even more complex lesions. Extended approaches may be considered in selected cases, and it allows the removal of a vascularized tumor or suprasellar residual tumor in order to avoid postoperative hematoma. The benefit offered by the extended endonasal route is to allow an extracapsular dissection of the tumor beside the standard endosellar corridor (3, 5, 35). Advancements in surgical route reconstruction techniques have contributed to ameliorate postsurgical outcome and performance status (36, 37).

Analyzing our metastasis series, three patients presented a pure intrasellar lesion (23%); four patients had intra-, supra-, and parasellar lesions (30%); and six patients had intra-, supra-, and retrosellar lesions (46%). Surgical strategy was tailored based on lesion extension, intraoperative histological examination, and performance status of the patients: in three cases, an extended endonasal approach was required; in five cases, only a biopsy was performed. The endoscopic endonasal standard approach was used in 10 patients; for the other three patients, an extended trans-planum approach was performed with good control of piecemeal resection.

Based on intraoperative histological examination, in the cases of intrasellar tumors, debulking could be smoother than supra- and retrosellar lesions, even if a poor performance status and a fibrous consistency with infiltrative pattern make biopsy a more reasonable choice. This principle is applied also in lesions extended to the supra-, para-, and retrosellar spaces in the cases which only a neurovascular decompression is possible (14) (15, 27, 30, 38).,

The proper management of a hypothalamic–pituitary metastasis requires a cogent balance between medical treatment, watchful waiting, surgery, and radiation therapy (1, 15, 23–25). Surgical resection can be complicated by fibrous consistency, irregular shape, and invasiveness of the tumor, which often lead to incomplete resections, increasing the risks of morbidity (27). Current literature shows one case report about a case of non-small cell lung cancer (NSCLC) with EGFR exon 19 deletion mutation, in which osimertinib eradicated the metastasis and prevented the need for radiation therapy (39).

In other studies, the main treatment for single brain metastasis is maximal safe surgical resection in combination with radio- and chemotherapy (16, 27, 30, 38, 40–42). In our series, this approach together with the combination of surgery and adjuvant therapy showed improvement in PFS and OS. Indeed, after multidisciplinary concertation, adjuvant therapy was used in all cases according to cogent protocols (24, 25). Ten patients were treated with systemic chemotherapy (59%), two patients (20%) had stereotactic radiotherapy on residual disease, and one patient with a clear cell renal carcinoma had a combination of radio- and chemotherapy. After adjuvant treatment, three patients (23%) developed pan-hypopituitarism.

In this study, the median progression-free survival and overall survival were 14 and 18 months, respectively (**Table 3**). Two patients are still alive 1 year after surgery. There were statistically significant differences in survival based on the type of adjuvant therapy on Kaplan–Meier analysis (i.e., radiotherapy was associated with a survival increase than chemotherapy). A limitation of this study is the reduced sample size. Before endonasal endoscopy introduction, the microsurgical transsphenoidal approach with partial resection and adjuvant treatment (local radiation) was associated with better symptom relief without effects on survival rates, which is less than 12 months in several studies; furthermore, the mean survival length in the clinical series was 6–7 months (28, 43–45). Zoli et al. reported a median survival of 11.8 months after transsphenoidal surgery followed by radiation therapy (46). In the series of anterior skull base metastases managed by the endoscopic endonasal approach reported by Zacharia et al., PFS and OS were 18 and 16 months and any correlation between survival and other variables was detected (41). In a similar multicentric study involving 12 patients, the mean OS was reported to be 17 months (30). The increase in survival is due to advancements in the surgical and oncological fields, and we do not speculate that only the endonasal approach has impacted survival.

**Table 3:** Surgical approach and reconstruction data.

Patient	Extended procedure	Reconstruction
**1**	−	NA
**2**	−	−
**3**	−	−
**4**	+	+
**5**	−	−
**6**	−	−
**7**	−	−
**8**	+	+
**9**	−	−
**10**	+	−
**11**	−	−
**12**	−	−
**13**	−	−

+ extended procedure, - not extended procedure; + osteo-dural reconstruction, - not osteo-dural reconstruction; NA information not available.

Concerning outcomes, recovery of the visual field defect and impairment of oculomotor nerve palsy were both observed in three of four patients (75%). Recovery of visual field defect was observed in six of seven patients with visual loss (85.7%), improvement of oculomotor nerve palsy occurred in four of seven patients with this defect (57.1%), while impairment of oculomotor nerve palsy was observed in three patients (42.9%). These results validate the role of endoscopic surgery as a tool for a satisfying decompression of the optic pathways. Regarding postsurgical complications, no postoperative cerebrospinal fluid leak occurred in any of the patients; one patient developed transient diabetes insipidus. According to current literature about the cases of pituitary metastasis managed with endoscopic endonasal surgery, this strategy is associated with a few complications and does not have an impact on the performance status of patients (1, 30, 34, 40, 41, 46).

A correct balance between surgical indications and evaluation of functional recovery impact on quality of life is mandatory. Indeed, not being able to know *a priori* if a lesion is metastatic or not, presurgical workup combined with surgical endoscopic experience has allowed a better interpretation of intraoperative features guiding the diagnosis and the subsequent management of the lesions. Furthermore, an extemporaneous histological examination is crucial to determine the surgical procedure and level of resection for improving PFS and OS (24, 25, 41).

## Conclusions

Pituitary metastasis surgery requires a cogent balance between medical treatment, watchful waiting, surgery, and radiation therapy; it requires cleverness, great versatility, and the collaboration of different specialists.

The endoscopic endonasal approach is a viable approach for the management of hypothalamic–pituitary metastases as this surgery provides adequate opportunity to obtain tissue sample and neurovascular decompression, both being crucial for continuing the integrated adjuvant therapy protocols.

## Data availability statement

The datasets presented in this study can be found in online repositories. The names of the repository/repositories and accession number(s) can be found in the article/supplementary material.

## Ethics statement

This study was approved by the institutional review board of the School of Medicine of University of Naples “Federico II”, which waived the need for informed consent due to the retrospective nature of the study. Written informed consent was obtained from the patients prior than any invasive clinico-diagnostic and surgical procedure; indeed, it was obtained for the eventual publication – for scientific purpose - of any patient records/information anonymously. The patients/participants provided their written informed consent to participate in this study.

## Author contributions

CB: writing of the original draft, review, and editing. TS, DS, and LC: revisions. RF and PCr: responsible for the pathology methods section; MDC, RF, and PC: acquisition of data. MS: statistic analysis. DS, FE, LC, and PCa: supervision and validation. All authors had full access to all the data in the study and take responsibility for the integrity of the data and the accuracy of the data analysis. All authors contributed to the article and approved the submitted version.

## Conflict of interest

The authors declare that the research was conducted in the absence of any commercial or financial relationships that could be construed as a potential conflict of interest.

## Publisher’s note

All claims expressed in this article are solely those of the authors and do not necessarily represent those of their affiliated organizations, or those of the publisher, the editors and the reviewers. Any product that may be evaluated in this article, or claim that may be made by its manufacturer, is not guaranteed or endorsed by the publisher.
